# Supplementary material for: Delaying an Electromagnetic Pulse with a Reflective High-Integration Meta-Platform
Source: Nanomaterials (Basel). 2024 Sep 3;14(17):1438. doi: 10.3390/nano14171438 (PMC11397699; doi:10.3390/nano14171438)
Supplement: Supplementary file 1 [file nanomaterials-14-01438-s001.zip › nanomaterials-3147060-supplementary.pdf]

Supplementary Materials

Delaying an Electromagnetic Pulse with a Reflective High-Integration Meta-Platform

Liangwei Li [1,2,†], Weikang Pan [1,2,†], Yingying Wang [1,2], Xiangyu Jin [1,2], Yizhen Chen [1,3], Zhiyan Zhu [1,2], Muhan Liu [1,2], Jianru Li [1,2], Yang Shi [1,2], Haodong Li [4,5,6], Shaojie Ma [1], Qiong He [4,5,6], Lei Zhou [4,5,6,*] and Shulin Sun [1,2,5,*]

[1] Shanghai Engineering Research Centre of Ultra Precision Optical Manufacturing, Department of Optical Science and Engineering, School of Information Science and Technology, Fudan University,
Shanghai 200433, China; 21210720008@m.fudan.edu.cn (L.L.); 19110720004@fudan.edu.cn (W.P.); 21210720013@m.fudan.edu.cn (Y.W.); 22210720009@m.fudan.edu.cn (X.J.); chenyz@lut.edu.cn (Y.C.); 21110720021@m.fudan.edu.cn (Z.Z.); 21110720013@m.fudan.edu.cn (M.L.); 23110720008@m.fudan.edu.cn (J.L.); 22110720011@m.fudan.edu.cn (Y.S.); shaojiema@fudan.edu.cn (S.M.)

[2] Yiwu Research Institute, Fudan University, Chengbei Road, Yiwu, 322000, China

[3] School of Science, Lanzhou University of Technology, Lanzhou 730050, China

[4] State Key Laboratory of Surface Physics (Ministry of Education), Fudan University, Shanghai 200433, China; 19110190007@fudan.edu.cn (H.L.); qionghe@fudan.edu.cn (Q.H.)

[5] Key Laboratory of Micro and Nano Photonic Structures (Ministry of Education), Fudan University, Shanghai 200433, China;

[6] Collaborative Innovation Center of Advanced Microstructures, Nanjing 210093, China

[*] Correspondence: phzhou@fudan.edu.cn (L.Z.); sls@fudan.edu.cn (S.S.)

[†] These authors contributed equally to this work.

Section A. Transient analysis of effective media model

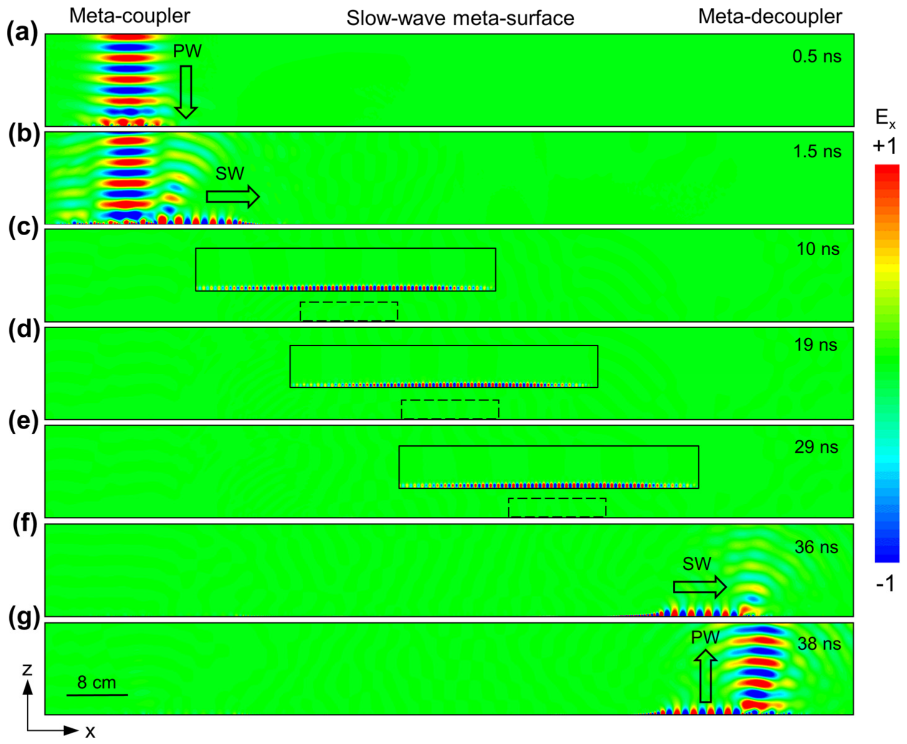

Figure S1. Calculated E-field (Re(E_x)) distribution inside the meta-device based on the effective media model at different times. Here, the incident Gaussian beam at the central frequency of 12. 975 GHz is just excited at (a) and converted to SWs at (b). Next, they are gradually slowed down to travel on the slow-wave meta-surface as depicted in (c-e). Eventually, the SWs are decoupled back to free space propagation waves (PWs) as shown in (f-g). In the whole process, the EM beam is totally delayed for approximately 36 ns. The efficiency of the device, defined by the ratio of the output power to the incident power, is about 73% according to our simulations in lossless case.

Section B. Design of the slow-wave meta-surface

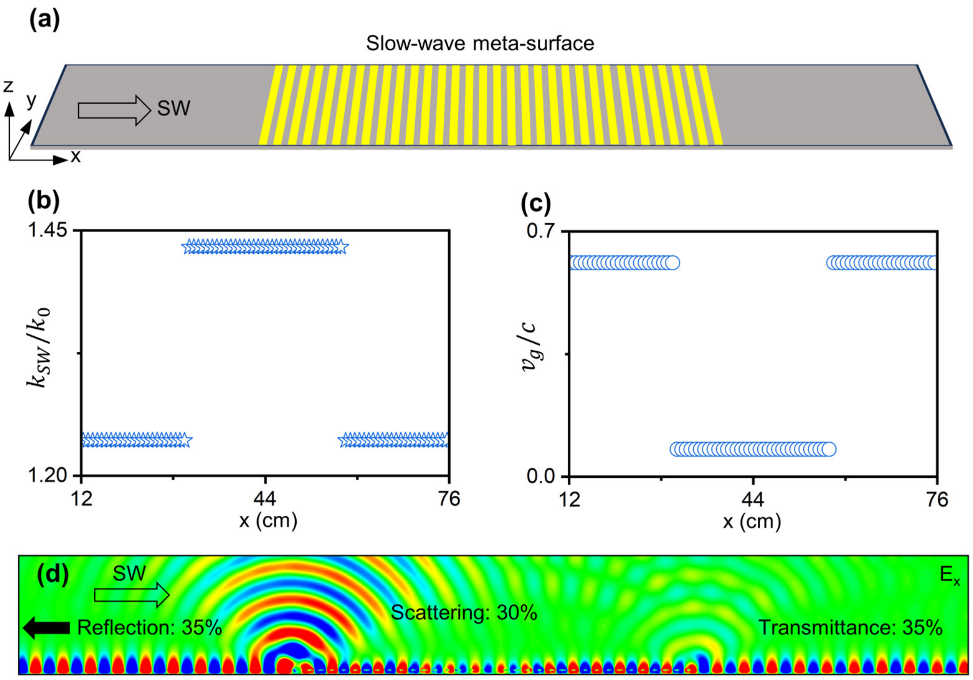

Figure S2. Slow-wave meta-surface with impedance mismatch at the two edges. (a) Schematic of the slow-wave meta-surface designed with the abruptly changed impedance at the two edges, where the patch width changes from 0 mm to 3.5 mm directly. (b, c) The distributions of the eigen wavevector and group velocity of SWs on such meta-surface. (d) Simulated E-field (E_x) distributions inside such slow-wave meta-surface excited by the SWs traveling along x direction. It is noted that the transmission efficiency is only about 35%. Due to the strong impedance mismatch at the two edges, there exists strong scattering and reflection losses (about 30% and 35%) in the device [1,2].

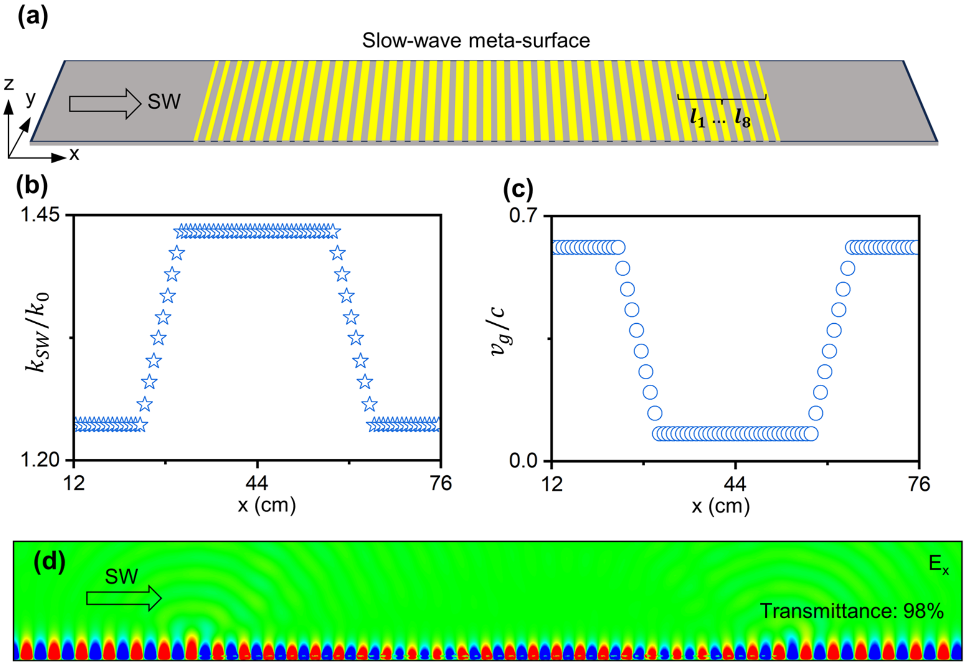

Figure S3. Slow-wave meta-surface with the slowly varied impedance at two edges. (a) Schematic of the slow-wave meta-surface with the slowly varied impedance at two edges, where the patch width has the gradient distributions of 2.1 mm, 2.5 mm, 2.74 mm, 2.92 mm, 3.1 mm, 3.21 mm, 3.33 mm, 3.42 mm. (b-c) The distributions of the eigen wavevector and group velocity of SWs on such meta-surface. (d) Simulated E-field (E_x) distributions inside such slow-wave meta-surface excited by the SWs traveling along x direction. Thanks to the gradient change of the effective impedance, both the scattering and reflection losses are significantly suppressed, giving rise to a high efficiency of about 98% [3,4].

Section C. Comparison of EM pulse delay for three different devices

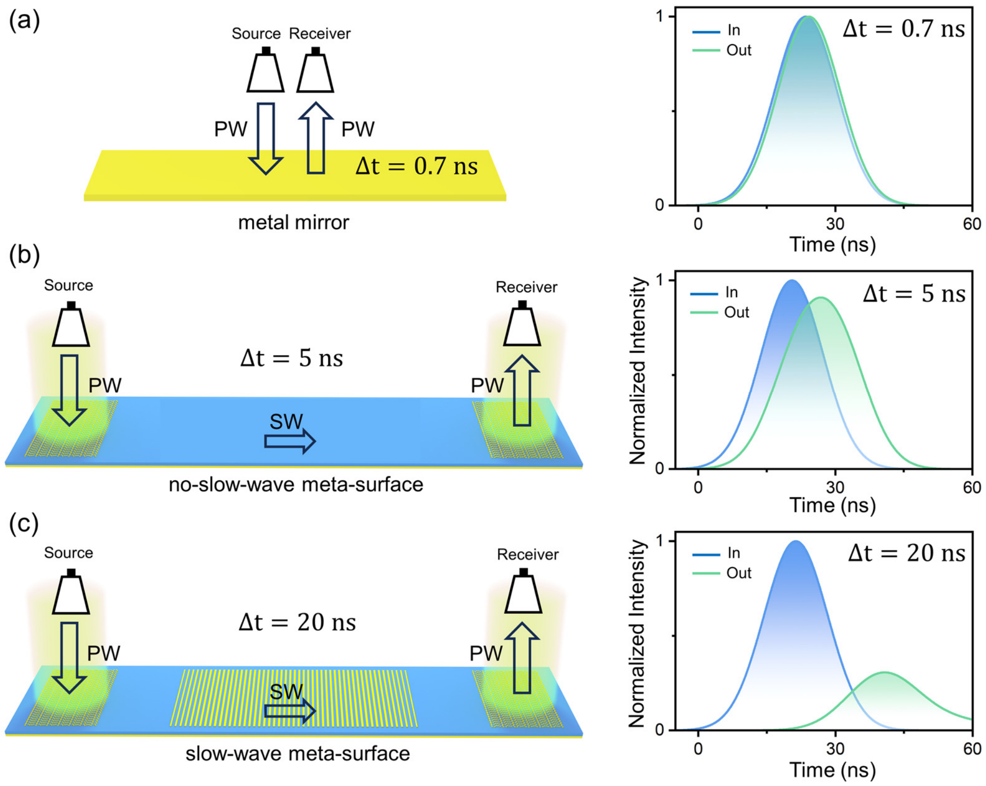

Figure S4. Comparison of EM pulse delay for three different devices: (a) a metallic mirror, (b) a high-integration meta-device composed of a meta-coupler, a homogeneous plasmonic metal (l=0 mm), and a meta-decoupler, and (c) a high-integration meta-device composed of a meta-coupler, a slow-wave meta-surface, and a meta-decoupler (corresponding to the device in Fig. 4a of the main text). Here, the frequency is fixed at 12.985 GHz.

Here, we compare the time delay effects of electromagnetic pulse based on three different systems: a metallic mirror, a high-integration meta-device composed of a meta-coupler, a homogeneous plasmonic metal (l=0mm), and a meta-decoupler, and a high-integration meta-device composed of a meta-coupler, a slow-wave meta-surface, and a meta-decoupler. In all three cases, the source antenna and receiver antenna are placed at 10 cm above the sample. As shown in Figure S4a, when an incident wave strikes the metallic mirror, the reflected wave is directly reflected back with nearly zero delay time.

Figure S4b shows that, when the propagating wave is incident on the meta-device without the slow-wave meta-surface, the time delay of the outgoing wave can also reach 5 ns originating from the propagation process of SW on the device. It should be noted that the velocity of SW, being about 0.61c, is only slightly smaller than free space light speed. Furthermore, when the wave is incident on meta-device with the slow-wave meta-surface, the delay time increases to 20 ns (see Figure S4c). Here, the frequency is fixed at 12.985 GHz in these studies.

Section D. Working efficiency and delay time of the proposed high-integration meta-device

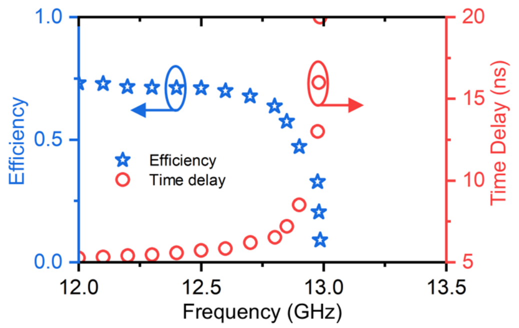

Figure S5. Working efficiency (blue stars) and delay time (red circles) of the high-integration meta-device corresponding to Figs. 4-6 of the main text as a function of frequency.

Section E. Numerical demonstration of an optical integrated meta-surface

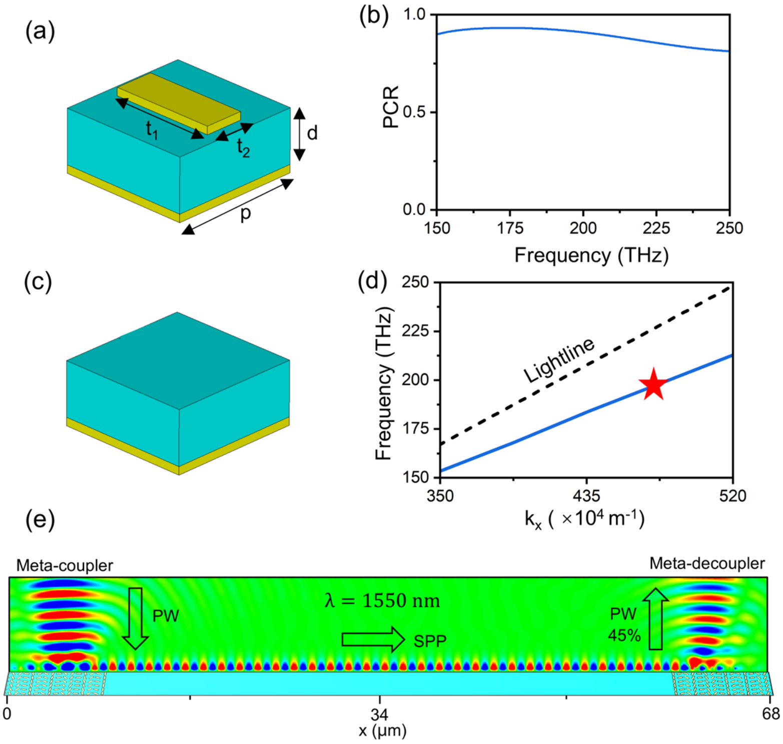

Figure S6. Numerical demonstration of an optical integrated meta-surface. (a) Schematic of the designed PB meta-atoms in metal-insulator-metal configuration for creating the optical meta-coupler and meta-decoupler. The geometric parameters are listed here: t_1=400 nm, t_2=150 nm, d=200 nm, p=500 nm, and the thickness of both top metallic bar and the bottom metallic film was set to be 30 nm. (b) Calculated spectra of the polarization conversion ratio (PCR) of the proposed PB meta-atoms, exhibiting high performance within a broad band. (c) Schematic of the building block of plasmonic metal that supports eigen SPP mode at optical frequency. Such a device can be easily obtained by simply removing the top metallic bars of the meta-atoms shown in (a). (d) Dispersion relation of the SPP supported by the plasmonic metal shown in (c). Here, the dash line represents the light line in vacuum. The eigen wavevector of SPP at 1550nm (red star) is about 1.14k_0 (k_0: total wavevector of light in vacuum). (e) Numerical demonstration of the PW-SPP-PW conversion based on the integrated meta-device, which consists of a PB meta-coupler (left), the homogenous plasmonic metal (middle), and the PB meta-decoupler (right). The orientation angles of two adjacent PB meta-atoms in meta-coupler and meta-

decoupler have a constant difference of 66.25°. Full-wave simulations demonstrate that the conversion efficiency and delay time in such coupling-decoupling system at the working wavelength of 1550nm are about 45% and 0.3 ps, respectively. Here, the metal is chosen as Ag with its permittivity described by the Drude model [5], and the dielectric spacer is SiO_2 with the permittivity of 2.09. If the slow-wave meta-surface is further integrated inside the present device, the delay time can be further increased. Besides, the working efficiency can be also improved by utilizing low-loss optical modes like waveguide modes supported by the conventional photonic integrated circuits.

References

1. Povinelli, M.L.; Johnson, S.G.; Joannopoulos, J.D. Slow-Light, Band-Edge Waveguides for Tunable Time Delays. *Opt Express* **2005**, *13*, 7145, doi:10.1364/OPEX.13.007145.
2. Guan, F.; Sun, S.; Ma, S.; Fang, Z.; Zhu, B.; Li, X.; He, Q.; Xiao, S.; Zhou, L. Transmission/Reflection Behaviors of Surface Plasmons at an Interface between Two Plasmonic Systems. *Journal of Physics: Condensed Matter* **2018**, *30*, 114002, doi:10.1088/1361-648X/aaad2a.
3. Lee, H.; Chen, T.; Li, J.; Painter, O.; Vahala, K.J. Ultra-Low-Loss Optical Delay Line on a Silicon Chip. *Nat Commun* **2012**, *3*, 867, doi:10.1038/ncomms1876.
4. Zhu, B.; Tang, S.; Zhou, L. Effective Medium Theory for Subwavelength Cylinder Arrays. *EPL (Europhysics Letters)* **2016**, *113*, 48002, doi:10.1209/0295-5075/113/48002.
5. Sun, S.; Yang, K.-Y.; Wang, C.-M.; Juan, T.-K.; Chen, W.T.; Liao, C.Y.; He, Q.; Xiao, S.; Kung, W.-T.; Guo, G.-Y.; et al. High-Efficiency Broadband Anomalous Reflection by Gradient Meta-Surfaces. *Nano Lett* **2012**, *12*, 6223–6229, doi:10.1021/nl3032668.
